# Surgical versus Transvalvular Aortic Valve Replacement in Elderly Patients—The Impact of Frailty

**DOI:** 10.3390/diagnostics11101861

**Published:** 2021-10-10

**Authors:** Andra Oancea, Cristina Furnică, Raluca Ozana Chistol, Florin Mitu, Maria-Magdalena Leon-Constantin, Grigore Tinică

**Affiliations:** 1 Faculty of Medicine, Grigore T. Popa University of Medicine and Pharmacy, 700115 Iasi, Romania; andra.oancea09@yahoo.com (A.O.); ralucachistol@gmail.com (R.O.C.); mitu.florin@yahoo.com (F.M.); leon_mariamagdalena@yahoo.com (M.-M.L.-C.); grigoretinica@yahoo.com (G.T.); 2 Department of Internal Medicine, Sf Spiridon University Hospital, 700111 Iasi, Romania; 3 Institute of Forensic Medicine, 700455 Iasi, Romania; 4 Cardiovascular Diseases Institute, 700503 Iasi, Romania; 5 Clinical Rehabilitation Hospital, Cardiovascular Rehabilitation Clinic, 700661 Iasi, Romania

**Keywords:** aortic stenosis, frailty, transcatheter aortic implantation, surgical aortic valve replacement

## Abstract

(1) Background: The aging process leads to an increased number of patients with cardiovascular diseases that require surgical treatment. One of the most common heart diseases with an increased prevalence in the elderly is aortic stenosis (AS). Recently, transcatheter aortic valve implantation (TAVI) has become the preferred technique for frail patients with high surgical risk. Currently, there is no gold standard method for assessing frailty. The available scores are objective, but limited by the lack of prospective information, especially from patients undergoing cardiac surgery or interventional procedures. Moreover, the current frailty scores record only certain aspects of the frailty identified in some system and organs. The aims of this study were to evaluate the different profile patients treated with TAVI or with surgical aortic valve replacement (SAVR) and to analyze the risk factors of unfavorable outcomes in the two groups to identify potential factors for frailty that could be included in the new scoring system. (2) Methods: This study included patients over 65 years old evaluated with severe, symptomatic AS treated with TAVI or SAVR admitted to the Cardiovascular Diseases Institute, Iasi. (3) Results: The group included 88 patients treated with TAVI compared with 218 patients undergoing SAVR. Most of the TAVI patients (84.09%) were over 70 years compared to 30.09% of the SAVR group. The TAVI patients had an increased surgical risk assessed by EuroSCORE II (15 vs. 4%) and severe heart failure (NYHA IV, 36.36 vs. 6.48%). The only residual symptom was dyspnea, in a significantly lower prevalence compared to preoperative evaluation. (4) Conclusions: TAVI improves the general status with results comparable to SAVR in elderly patients with increased severity of the disease and higher prevalence of comorbidities.

## 1. Introduction

The prevalence of aortic stenosis (AS) is increasing due to the growing elderly population, reaching approximately 5% of patients over 65 years in the USA. Osnabrugge et al., in a meta-analysis conducted in Europe, the USA, and Taiwan, found a prevalence of AS of 12.4%, while in patients over 75 years, the prevalence of severe AS was 3.4% [1]. The prevalence of AS increases exponentially with age, from 0.2% in patients between 50 and 59 years, to approximately 10% in patients over 80 years [2]. Most patients with AS are asymptomatic for a long period of approximately 10–20 years, but after the onset of symptoms, life expectancy decreases dramatically, respectively five years for angina, three years in the case of syncope, and two years if heart failure develops [3]. Untreated, patients with severe AS have a life expectancy of 64% at one year, 25% at three years, and 3% at six years [4].

Transcatheter aortic valve implantation (TAVI) opened a new era in the therapeutic management of severe and symptomatic AS in patients with high operative risk, especially in the elderly. The ESC/EACTS Guidelines for the management of valvular heart disease recommends using frailty as a criterion in the selection of patients for TAVI procedures [5]. Given the profile of diseases, the prevalence of frailty is high in patients with cardiovascular diseases and burdened by a high prognostic significance. Collard et al., in an analysis of 61,500 older adults from developed countries, observed that approximately one in ten adults over 65 years is frail [6]. In a review of nine studies, including 52,250 patients followed over a period of 6.2 years, a 4.1 fold higher risk of frailty among cardiovascular patients was demonstrated [7]. There are numerous measurement methods for the screening, diagnostic, and quantification of frailty severity, but there is no consensus on the definition and the parameters that should be included in the evaluation. Fried et al. described frailty by the presence of a minimum of three of the following physical characteristics: Weight loss, exhaustion, low physical activity, slow walking speed, and low handgrip strength [8]. The Short Physical Performance Battery (SPPB) is an alternative for assessing frailty using three parameters (speed walking, ability to get up from a chair, and balance) evaluated on a scale from 0 to 4 [9]. In contrast, the Frailty Index described by Rockwood et al. considers frailty through the presence of the accumulation of multidimensional deficits [10]. The questionnaire is based on a series of signs, symptoms, disabilities, and laboratory investigations in several medical fields.

### Study Purpose

The primary purpose of the present study was to evaluate the different profiles of patients with severe aortic stenosis undergoing SAVR and TAVI. The secondary purpose was to analyze the risk of unfavorable outcomes in the two groups in order to identify the potential factors that could be included in a new scoring system.

## 2. Materials and Methods

### 2.1. Study Population

A total of 306 patients evaluated with AS with indications of valve prosthesis were included in the study, of which 88 patients undergoing TAVI and 218 patients with isolated SAVR.

Aortic valve replacement was indicated in patients with severe AS with a mean gradient > 40 mmHg or a jet velocity > 4 m/s and an aortic valve area < 0.8 cm^2^. In cases <65 years with a life expectancy > 20 years and no prohibitive surgical risk, SAVR was preferred. TAVI was indicated following a multidisciplinary evaluation in accordance with the recommendations of the 2017th edition of the European Cardiology Society Guide to patients aged > 65 years, with a high or prohibitive surgical risk (Society of Thoracic Surgeons score or EuroSCORE II ≥ 4% or logistic EuroSCORE I ≥ 10%, frailty, porcelain aorta, and sequelae of thoracic irradiation), a life expectancy < 10 years but >1 year, and a chance of over 25% to survive with benefits for two years after the procedure.

The aortic valve and root were evaluated by echocardiography and cardiac computer tomography (CT) in all cases. Medical imaging investigations were used to confirm the diagnostics of aortic stenosis, to select the prosthesis size, to assess the implantation angle, to estimate implantation difficulties, and to choose the access route.

All interventions were performed under conditions of general anesthesia, with orotracheal intubations, blood pressure monitoring, and central jugular venous access. Postoperatively, patients were supervised in the intensive care unit.

Patients who required concomitant repair or replacement for other valve diseases (e.g., aortic regurgitation, mitral, or tricuspid stenosis and regurgitation) or other cardiovascular diseases (e.g., coronary artery bypass graft) were excluded from the study.

### 2.2. Ethics Approval

Written informed consent of patients was obtained in all cases and allowed us to review the observation sheets and operative protocols and to use the data in the present research. The study was performed in accordance with the Romanian medical legislation and approved by the Ethics Commission of the University of Medicine and Pharmacy “Grigore T. Popa” Iasi (certificate of approval from 15 June 2020) and Cardiovascular Diseases Institute, Iasi.

### 2.3. Statistical Methods

The information obtained from the observation sheets and surgical protocols of the selected patients were entered into an Excel database using Microsoft Excel 365 for Microsoft Windows 10 and was statistically analyzed using the Medcalc 19.5.2 program. The results are presented as tables, graphical representations, and dedicated statistical tests. Continuous variables are expressed as means ± standard deviations. Categorical variables are expressed as total number of cases and percentages. Groups were compared using the *χ*^2^ or Fisher exact test for categorical variables and ANOVA/Student’s *t*-test and Wilcoxon–Mann–Whitney U/Kruskal Wallis tests for continuous variables based on their distribution. Normality was checked using the Kolmogorov–Smirnov and Shapiro–Wilk W tests. The association of the different variables was verified with the Pearson’s correlation test.

## 3. Results

### 3.1. Patient Characteristics

The baseline demographic, clinical, and periprocedural characteristics of patients are presented in Table 1.

Compared to SAVR patients, the TAVI group were, on average, nine years older, 84.09% of them being over 70 years old compared to 30.09% of those treated with SAVR. Moreover, TAVI patients had a higher comorbidity burden. As can be seen from Table 1, the TAVI patients had an increased surgical risk assessed by EuroSCORE II (15 vs. 4%), severe heart failure (NYHA IV, 36.36 vs. 6.48%), and pulmonary hypertension (25 vs. 5.09%). The values of LVEF were lower in the TAVI group (54.13 ± 10%) compared to the SAVR cohort 55.41 ± 9.14%. Additionally, the TAVI patients registered a higher comorbidity burden, such as previous myocardial infarction (11.36 vs. 1.29%), stroke (6.82 vs. 5.96%), COPD (14.77 vs. 11%), and PAD (13.64 vs. 4.17%).

### 3.2. Early Postpreocedural Evolution

The length of postoperative hospitalization (intensive care and early recovery) was significantly shorter in the TAVI group (*p* < 0.001), respectively 9.71 ± 7.61 days compared to 16.78 ± 8.56 days in the SAVR group. Given the fact that the patients received blood and blood derivates, the hemoglobin value between the two groups did not register significant differences early after surgery (11.16 vs. 11.13 mg/dL). LVEF remained stable in both groups (53% in the TAVI group vs. 54% in the SAVR group), similar to the preoperative values. The implanted prostheses were normo-functional, both in the TAVI and SAVR patients, with a significant decrease in the maximum trans-valvular gradient from 89 ± 27 mmHg to 21.77 ± 10.89 mmHg in the case of TAVI and 88.49 ± 25.20 mmHg to 24.39 ± 8.29 mmHg for SAVR. Mild and moderate prosthetic regurgitation was identified in 27 cases, 24 patients from the TAVI group (27.27%) and three patients from the SAVR group (1.39%), without clinical significance. No cases of early prosthesis dysfunction were identified.

Regarding general complications, the incidence was similar in the two groups, except for postoperative arrhythmias (e.g., atrial fibrillation), which was more frequent in the SAVR cohort (Table 2). Only four patients of the TAVI group (4.54%) developed atrioventricular block that required permanent pacemaker implantation.

A total of nine patients (five with TAVI and four with SAVR) deceased within 30 days from the intervention because of acute complications (renal failure, sepsis, and upper gastrointestinal hemorrhage). Only two TAVI patients deceased because of specific complications, one case of aortic dissection and one case of annular rupture.

### 3.3. Thirty-Day Evaluation

Thirty-day evaluation was available in 128 cases, 42 patients from the TAVI group and 86 patients from the SAVR cohort. In the context of the significant age difference and the prevalence of comorbidities, the postprocedural clinical benefit was similar in both groups. Angina and syncope were not reported during the 30 day evaluation by any of the patients with TAVI, only dyspnea persisted in 19.05% of cases, but given the multifactorial etiology of this symptom, it is difficult to attribute to aortic pathology, especially in elderly patients (Table 3). The severity of heart failure decreased significantly, the benefit being more important in the TAVI cohort, where 28.57% of patients were in the NYHA class IV before the procedure.

The LVEF improved slightly by the 30-day follow up compared to preoperative status in the TAVI group (from 53.69 ± 10.62% to 54.46 ± 9.12%) comparative to the SAVR group, in which there were no changes (from 56.28 ± 8.79% to 56.14 ± 8%). Administration of an iodinated contrast agent did not significantly alter renal function by the 30-day evaluation in the TAVI patients (serum creatinine 9.95 ±. 2.37 mg/dL at 30-day follow-up vs. 1.07 ± 0.24 mg/dL before the intervention). The prostheses were normo-functional at one month in both groups, with a peak transvalvular gradient of 18.59% ± 5.77 mmHg in the TAVI group and 22.09 ± 8.99 mmHg in the SAVR group. A slight, insignificant regurgitation was encountered in six cases, three from the TAVI group and three from the SAVR group.

### 3.4. Postprocedural Evaluation at Six Months

A total of 61 patients presented at the six months postprocedural evaluation, 29 patients from the TAVI group and 32 patients from the SAVR group. Dyspnea was the only persistent symptom after the procedure, without any significant worsening compared to the 30-day assessment. Heart failure continued to improve significantly between the 30-day and six-month evaluations in both groups; only two patients (6.89%) from the TAVI group were in NYHA class III. The LVEF, renal function, and functionality of the prostheses remained at similar values in the two groups between the evaluations. Valvular regurgitation progressed in nine cases. Only one of the three TAVI patients evaluated at 30 days with grade one aortic regurgitation progressed to grade two, while in the SAVR group, three patients developed mild regurgitation.

### 3.5. One-Year Evaluation

A total of 52 patients presented for control at one year after the intervention, nine TAVI patients and 43 patients from the SAVR group. In terms of symptoms, two patients (4.65%) from the SAVR cohort developed angina, while syncope was absent in both groups. The prevalence of dyspnea increased in the TAVI group (33.33%, three patients), while in the SAVR group, the prevalence was stationary (18.6%). The limited number of TAVI patients who presented at the 12-month evaluation did not allow a statistical test to be performed. Heart failure did not worsen between the two evaluations, with 90% of the patients in both groups in the NYHA class II (Table 4).

LVEF continued to remain stable at one year after the procedure, with mean values of 56.67 ± 5.1% in the TAVI cohort and 57.89 ± 4.67% in the SAVR group. The prosthesis did not show significant dysfunction, with a peak transvalvular gradient of 22.33 mmHg in the TAVI group and 24.46 mmHg in the SAVR group. Two of the TAVI patients were evaluated with valvular regurgitation, one with mild regurgitation and the other with moderate regurgitation. From the SAVR group, eight patients presented with mild regurgitation and one patient with moderate regurgitation.

### 3.6. Mid-Term Mortality

By the mid-term (>30 days), 26 patients were deceased—13 from the TAVI group (15.66%) and 13 from the SAVR group (6.07%). The profiles of these patients are presented in Table 5.

As can be noted, atrial fibrillation and CKD were present in five deceased patients in the TAVI group. In the SAVR group, PAD was the most frequently encountered comorbidity in deceased patients (four cases). The second most frequent comorbidity was diabetes mellitus in the TAVI group (three cases) and PAD in the SAVR group (four cases).

## 4. Discussion

In our study, we compared the short- and mid-term outcomes in patients undergoing either TAVI or SAVR, in order to identify demographical, clinical, and paraclinical factors that could influence post-procedural evolution and could be used as a potential parameter in a risk assessment scoring system in TAVI.

Van Mourik et al. performed a meta-analysis that included 49 observational studies and identified a series of preoperative factors characteristic of frailty with prognostic value in TAVI [11]. These factors are preoperative chronic lung disease, chronic kidney disease (CKD), underweight (BMI < 20 kg/m^2^), hypoalbuminemia, frailty according to frailty score, anemia, gait speed, and ADL (activities of daily living) independence. In our study group, the incidence of underweight patients was low, most being normal or overweight, which allowed us to exclude this factor.

Chronic lung diseases represent approximately 1/3 of TAVI candidates and are considered to have a major impact on the short- and long-term prognosis, especially in case of oxygen dependence and limited mobility. Takagi el al. performed a meta-analysis of six studies, which included 47,771 patients evaluated with COPD patients that underwent TAVI or SAVR [12]. Their analysis indicated that COPD patients undergoing TAVI had a significant lower early mortality rate compared to SAVR, while there was no significant difference in the mid-term mortality between the two procedures. In our group, 14.77% patients presented with COPD compared to 11% in the SAVR group. By the mid-term, one of the 13 TAVI patients that deceased presented with COPD; thus, we cannot affirm any association with mortality. CKD registered a higher prevalence rate in TAVI patients that deceased on the mid-term (five of the 13 cases) and was not present in any of the SAVR patients that deceased > 30 days postoperatively, confirming prior studies.

Although the low mortality rate in our groups did not allow us to perform a separate statistical analysis, we propose two additional factors that could have prognostic value for the mid-term outcome in TAVI patients in terms of mortality, namely, preoperative diabetes mellitus (three of the 13 deceased patients) and preoperative atrial fibrillation (five of the 13 deceased patients). These factors need further assessment, but we consider that a risk stratification model is required in TAVI to identify patients at risk in order to intensify pre- and postoperative care.

The cumulative mortality at one year in the TAVI group was 20.45% (18 deaths—5 in-hospital and 13 a while after surgery), significantly higher compared to the SAVR treated group—10.12% (17 deaths—four deaths in hospital and 13 a while after surgery). This two-fold higher mortality should not be attributed to the intervention, given the significant differences between the two cohorts. The TAVI patients were older than the SAVR group and had an important history of cardiovascular diseases (11.36% were previously diagnosed with myocardial infarction compared to 1.39% in the SAVR group; 13.64% were evaluated with PAD vs. 4.17% in the SAVR group).

The TAVI patients were referred to the clinic as a last resort, after years of medical treatment, the condition worsening in the meantime, with patients being considered ineligible for surgical treatment. Thus, we can speak of an iatrogenic increased risk that has not been analyzed by any specialized study, and we propose the inclusion of another parameter—the time since diagnosis and the establishment of treatment. We consider that this factor reflects the severity of the disease and can predict complications.

In terms of benefit of the intervention, the analysis of the follow-up evaluations revealed significant improvement, with an almost complete disappearance of symptoms. The only residual symptom was dyspnea, which is non-specific to the AS and has a multifactorial determinism that includes frailty and chronic lung diseases.

The success of the intervention was also certified by the continuous regression of the NYHA class; the percentage of patients in NYHA class II was 85.71% at the 30-day follow up, 93.10% at six months, and 88.98% at the one-year evaluation. The paraclinical evaluation reflected the clinical evolution. The LVEF remained above 50% in all three evaluations and the serum creatinine maintained at normal values of a maximum of 1.09 mg/dL. This reflects a negligible effect on the renal function of the iodinated contrast administrated during the TAVI intervention.

The aortic transvalvular gradient indicated a normo-functional status of implanted prosthesis with a maximum gradient of 23.69 ± 10.27 mmHg at 12 months. Valvular regurgitation was negligible in the TAVI group (three cases of grade one and two regurgitation).

In the settings of significantly older patients, namely, increased disease severity and higher prevalence of comorbidities and frailty, we can affirm that TAVI substantially improves patients’ status with results comparable to SAVR. The postprocedural mortality of TAVI patients is determined more by frailty syndrome than by the intervention itself. Finn et al. estimated that 80% out of the 102 high-risk patients were frail, which led to an increase in one-year mortality to 17% [13]. Puls et al. quantified the same mortality rate of 17% in frail patients, but one month after TAVI [14]. The one-year mortality was 56% compared to 24% in non-frail patients [13]. In our group, the mortality of 20.45% at one year after TAVI was registered, regardless of the level of frailty, the result being superior to those reported in the literature.

Osnabrugge et al. stated the absence of a benefit of TAVI in 39% of patients with high operative risk, a result that was not supported by our study, where the benefit was certified by clinical and paraclinical data [15].

The major limitation regarding the evaluation of the impact of frailty on the selection of patients and the timing intervention is represented by the absence of randomized studies comparing groups of frail and non-frail patients. All studies published up to date, including the present research, show an important bias represented by underlying and unquantified frailty.

## 5. Conclusions

The TAVI procedure significantly improves the status of patients with symptomatic, aortic stenosis with results comparable to SAVR in the setting of significantly older patients, increased disease severity, higher prevalence of comorbidities, and frailty. The short- and mid-term mortality recorded in the TAVI group were lower than that reported by other studies, attributable to comorbidities and frailty and not to specific complications of the intervention. We propose two additional factors to be studied, diabetes mellitus and atrial fibrillation, as potential risk factors of mid-term mortality after TAVI.

## Figures and Tables

**Table 1:** General characteristics of the patient cohorts.

Parameter	TAVI (88 Cases)	SAVR (218 Cases)	*p*-Value
Age (years)	76.1 ± 7.34	67.72 ± 5.79	<0.001
Gender (no., % female)	47 (53.41)	97 (44.49)	n.s.
BMI (kg/m^2^)	28.56 ± 4.05	28.15 ± 5.26	n.s.
EuroSCORE II	15%	4%	<0.001
AHT (no., %)	48 (54.55)	135 (62.5)	n.s.
NYHA (no., %)	NYHA I–0 (0) NYHA II–2 (2.27) NYHA III–54 (61.36) NYHA IV–32 (36.36)	NYHA I–7 (3.24) NYHA II–52 (24.07) NYHA III–143 (66.20) NYHA IV–14 (6.48)	<0.001
Hemoglobin (mg/dL)	12.60 ± 1.88	13.07 ± 1.53	0.049
Type 2 DM (no., %)	14 (15.91)	41 (18.98)	n.s.
MI (no., %)	10 (11.36)	3 (1.39)	0.0001
Stroke	6 (6.82)	13 (5.96)	n.s.
eGFR (mL/min)	78.33 ± 23.03	72.95 ± 15.83	n.s.
COPD (no., %)	13 (14.77)	24 (11)	n.s.
PAD (no., %)	12 (13.64%)	9 (4.17)	0.0021
Hepatitis (no, %)	6 (6.82)	9 (4.17)	n.s.
LVEF (%)	54.13 ± 10	55.41 ± 9.14	0.037
Maximum gradient (mmHg)	89 ± 27.40	88.49 ± 25.20	n.s.
TAPSE (mm)	22 ± 4.53	23.48 ± 3.61	n.s.
PPM (no., %)	5 (5.68)	3 (1.38)	n.s.
CABG (no., %)	8 (9.09)	0	0.0006
PH (no., %)	Grades 1–17 (19.32) Grades 2–19 (21.59) Grades 3–22 (25)	Grades 1–24 (11.01) Grades 2–26 (12.04) Grades 3–11 (5.09)	0.0089

BMI, body mass index; AHT, arterial hypertension; NYHA, New York Heart Association; DM, diabetes mellitus; MI, myocardial infarction; GFR, glomerular filtration ratio; COPD, chronic obstructive pulmonary disease; PAD, peripheral artery disease; LVEF, left ventricle ejection fraction; PPM, permanent pacemaker; CABG, coronary artery bypass graft; PH, pulmonary hypertension.

**Table 2:** Postoperative morbidity and mortality depending on the type of treatment.

Parameter	TAVI (88 Cases)	SAVR (218 Cases)	*p*-Value
Acute kidney injury	13 (14.77%)	24 (11.1%)	n.s.
Postoperative AMI	0 (0%)	0 (0%)	n.s.
Infectious complications	9 (10.23%)	16 (7.41%)	n.s.
Stroke/TIA	0 (0%)	1 (0.46%)	n.s.
Hemorrhagic complications	8 (9.09%)	18 (8.33%)	n.s.
Postoperative arrhythmias	24 (27.27%)	97 (44.91%)	0.003
PPM	4 (4.54)	0 (0)	n.s.
Death < 30 days	5 (5.68%)	4 (1.83%)	n.s.

**Table 3:** The 30-day clinical benefit.

	Angina		Syncope		Dyspnea	
	Preprocedural	30 Days	Preprocedural	30 Day	Preprocedural	30 Days
TAVI	21 (50%)	0%	4 (9.51%)	0%	40 (95.24%)	8 (19.05%)
SAVR	39 (45.35%)	1 (1.16%)	11 (12.9%)	0%	75 (87.21%)	14 (16.28%)

**Table 4:** Heart failure evolution.

	NYHA II				NYHA III	
	Preprocedural	30 Days	6 Months	Preprocedural	30 Days	6 Months
**TAVI (9 cases)**	2 (2.27%)	8 (88.89%)	8 (88.89%)	54 (61.36%)	1 (11.11%)	1 (11.11%)
**SAVR (43 cases)**	52 (24.07%)	41 (95.35%)	2 (4.65%)	143 (66.20%)	42 (97.67%)	1 (2.33%)

**Table 5:** Mid-term mortality.

TAVI (13 Cases)	SAVR (13 Cases)
Diabetes mellitus—3 cases	Sepsis—3 cases
Acute myocardial infarction (prior CABG)—1 case	AV block with PPM—1 case
Neoplasia—1 case	PAD—4 cases
CKD—5 cases	Viral hepatitis—2 cases
COPD—1 case	Atrial fibrillation—7 cases
PAD—1 case	Aortic regurgitation—1 case
Atrial fibrillation—5 casesPostprocedural sepsis—1 caseEuroSCORE—24.75%	Acute renal failure—2 cases


Acute renal failure—2 cases	
Aortic regurgitation—2 cases	

## Data Availability

The data presented in this study are available on request from the corresponding author.
